# The Dutch CAR-T Tumorboard Experience: Population-Based Real-World Data on Patients with Relapsed or Refractory Large B-Cell Lymphoma Referred for CD19-Directed CAR T-Cell Therapy in The Netherlands

**DOI:** 10.3390/cancers15174334

**Published:** 2023-08-30

**Authors:** Anne M. Spanjaart, Elise R. A. Pennings, Pim G. N. J. Mutsaers, Suzanne van Dorp, Margot Jak, Jaap A. van Doesum, Janneke W. de Boer, Anne G. H. Niezink, Milan Kos, Joost S. P. Vermaat, Aniko Sijs-Szabo, Marjolein W. M. van der Poel, Inger S. Nijhof, Maria T. Kuipers, Martine E. D. Chamuleau, Pieternella J. Lugtenburg, Jeanette K. Doorduijn, Yasmina I. M. Serroukh, Monique C. Minnema, Tom van Meerten, Marie José Kersten

**Affiliations:** 1 Department of Hematology, Amsterdam UMC Location University of Amsterdam, 1007 MB Amsterdam, The Netherlands; 2 Cancer Center Amsterdam, 1105 AZ Amsterdam, The Netherlands; 3 LYMMCARE (Lymphoma and Myeloma Center Amsterdam), 1105 AZ Amsterdam, The Netherlands; 4 Erasmus School of Health Policy and Management, Erasmus University Rotterdam, 3062 PA Rotterdam, The Netherlands; 5 Department of Hematology, Erasmus MC Cancer Institute, University Medical Center Rotterdam, 3015 GD Rotterdam, The Netherlands; 6 Department of Hematology, Radboud University Medical Center, 6500 HB Nijmegen, The Netherlands; 7 Department of Hematology, University Medical Center Utrecht, 3584 CX Utrecht, The Netherlands; 8 Department of Hematology, University Medical Center Groningen, 9713 GZ Groningen, The Netherlands; 9 Department of Radiation Oncology, University Medical Center Groningen, 9713 GZ Groningen, The Netherlands; 10 Department of Medical Oncology, Amsterdam UMC Location University of Amsterdam, 1081 HV Amsterdam, The Netherlands; 11 Department of Hematology, Leiden University Medical Center, 2333 ZA Leiden, The Netherlands; 12 Department of Internal Medicine, Division of Hematology, GROW School for Oncology and Developmental Biology, Maastricht University Medical Center, 6229 HX Maastricht, The Netherlands; 13 Department of Hematology, Amsterdam UMC Location Vrije Universiteit Amsterdam, 1081 HV Amsterdam, The Netherlands; 14 Department of Internal Medicine-Hematology, St Antonius Hospital, 3435 CM Nieuwegein, The Netherlands

**Keywords:** CAR T-cell therapy, real-world data, outcomes, LBCL

## Abstract

**Simple Summary:**

CAR T-cell therapy has emerged as the new standard of care for patients with relapsed/refractory (R/R) large B-cell lymphoma (LBCL), but real-world outcomes differ across countries. Additionally, real-world data on health-related quality of life (HR-QoL) are scarce but important, as they reflect the direct experience of patients. In the Netherlands, patients can be referred to the CAR-T tumorboard, a national CAR-T expert panel, who decide whether CAR-T is a feasible treatment option. This multicenter study reports on the favorable outcomes, including the HR-QoL, of axicabtagene ciloleucel (axi-cel) for patients with R/R LBCL after ≥2 lines of systemic therapy in the Netherlands. On the other hand, we show that a substantial proportion of patients are still in need of alternative treatments, including improved CAR-T strategies, as they are unfit for or do not respond to axi-cel. Comparing real-world outcomes between cohorts could help to select best practices and further optimize CAR-T treatment.

**Abstract:**

The real-world results of chimeric antigen receptor T-cell (CAR-T) therapy for patients with relapsed/refractory (R/R) large B-cell lymphoma (LBCL) substantially differ across countries. In the Netherlands, the CAR-T tumorboard facilitates a unique nationwide infrastructure for referral, eligibility assessment and data collection. The aim of this study was to evaluate real-world outcomes of axicabtagene ciloleucel (axi-cel) in the Dutch population, including the thus-far underreported effects on health-related quality of life (HR-QoL). All patients with R/R LBCL after ≥2 lines of systemic therapy referred for axi-cel treatment between May 2020–May 2022 were included (N = 250). Of the 160 apheresed patients, 145 patients received an axi-cel infusion. The main reason for ineligibility was rapidly progressive disease. The outcomes are better or at least comparable to other studies (best overall response rate: 84% (complete response: 66%); 12-month progression-free-survival rate and overall survival rate: 48% and 62%, respectively). The 12-month NRM was 5%, mainly caused by infections. Clinically meaningful improvement in several HR-QoL domains was observed from Month 9 onwards. Expert-directed patient selection can support effective and sustainable application of CAR-T treatment. Matched comparisons between cohorts will help to understand the differences in outcomes across countries and select best practices. Despite the favorable results, for a considerable proportion of patients with R/R LBCL there still is an unmet medical need.

## 1. Introduction

CD19-directed chimeric antigen receptor T-cell (CAR-T) therapy has become the standard of care for patients with relapsed/refractory (R/R) large B-cell lymphoma (LBCL). Prior to the introduction of CAR-T, patients with R/R LBCL after two or more lines of systemic therapy had a very poor prognosis with a lack of curative treatment options [1]. The pivotal phase-2 trials ZUMA-1, JULIET and TRANSCEND evaluated treatment for these patients with axicabtagene ciloleucel (axi-cel), tisagenlecleucel (tisa-cel) and lisocabtagene maraleucel (liso-cel), respectively, and showed unprecedentedly high response rates and durable remissions in 30–40% of patients [2,3,4,5,6,7]. This resulted in the US Food and Drug Administration and European Medicines Agency (EMA) approvals of all three CD19-CAR-T products for R/R LBCL after ≥2 lines of systemic therapy. The US consortia with extensive CAR-T trial experience were the first to show that these impressive results were reproducible in routine clinical practice, even in patients not meeting the eligibility criteria used in the pivotal trials [8,9,10]. More recently, real-world outcomes in European cohorts were also published [11,12,13,14]. However, these real-world outcomes of CAR-T infused patients substantially differ across cohorts; and for some European cohorts, the outcomes are less favorable compared to results in the US [15]. Although a more diverse patient population is being treated in the real-world setting, only a selected subset of patients meeting the product registration label is considered fit enough to receive CAR-T therapy. The selection procedure to determine which patients are fit for CAR-T differs across and sometimes within countries and might influence the outcomes. The exact proportion of the entire R/R LBCL patient population fulfilling the product registration label that is not selected for CAR-T is unknown, due to a paucity of population-based studies. To better understand differences in outcomes and to select best practices, studies reporting on the selection procedure and all other important aspects of the complete treatment trajectory, such as bridging strategies and time between referral, leukapheresis and infusion are needed. Furthermore, the impact on the health-related quality of life (HR-QoL) in the real world is currently underreported, while preserving or improving HR-QoL is key and could play an important role in guiding treatment choices with novel therapeutic options, such as bispecific antibodies, becoming available [16,17]. In the Netherlands, nationwide referral, eligibility assessment and data collection for CAR-T are centralized in a national tumorboard and registry (“Follow that CAR!”). The Dutch CAR-T tumorboard was established in May 2020 when the first CAR-T product (axi-cel) became commercially available in the Netherlands, with the ambition to optimize (cost-)effective, high-quality CAR-T care. All Dutch centers qualified for CAR-T treatment for adults (N = 8) are represented by CAR-T experts in the tumorboard, who discuss CAR-T eligibility and treatment strategies for referred patients in a virtual meeting taking place twice a week. This multicenter cohort study reports on the first two years of the commercial use of axi-cel in the Netherlands and aims to evaluate population-based real-world data on patients with R/R LBCL referred for CD19-CAR-T treatment from the time of referral to the national tumorboard, including the selection procedure, bridging strategies, time between referral, leukapheresis and infusion and HR-QoL.

## 2. Materials and Methods

### 2.1. Study Design and Study Population

All adult patients (age ≥ 18 years) referred to the Dutch CAR-T tumorboard for treatment with commercially available CD19-CAR-T for R/R LBCL after ≥2 lines of systemic therapy between May 2020–May 2022 were evaluated. In the Netherlands, tisa-cel was reimbursed for this indication only as of June 2022 and liso-cel is not reimbursed; thus, only axi-cel was evaluated. Referred patients can be divided into 3 subgroups: (1) “screening-only” (i.e., patients screened for axi-cel treatment who did not undergo leukapheresis), (2) “apheresis-only” (i.e., patients who underwent leukapheresis but did not receive axi-cel infusion), and (3) “infused” (i.e., patients who received an axi-cel infusion). The eligibility criteria used by the tumorboard between May 2020 and May 2022 are provided in the Supplementary S1. Only adult patients with R/R LBCL (diffuse large B-cell lymphoma (DLBCL; including transformed follicular lymphoma (tFL) and high-grade B-cell lymphoma (HGBCL)) and primary mediastinal large B-cell lymphoma (PMBCL)) after ≥2 lines of systemic therapy willing to be treated with axi-cel (i.e., appropriate indication as per product registration label) were included in this multicenter cohort study for further analyses. For these patients, data on time paths, bridging strategies, efficacy and toxicity were retrospectively collected from tumorboard referral forms and medical records at all Dutch CAR-T treating centers with a data cut-off date of 1 May 2023. HR-QoL data were collected in 3 CAR-T treating centers at baseline (before CAR-T infusion) and follow-up at Month 1, 3, 6, 9 and 12 post-infusion using paper versions of two validated questionnaires, the EORTC QLQ-C30 and the EQ-5D-5L. The study was conducted in accordance with the Declaration of Helsinki and approved by the Medical Ethics Review Committee of the Academic Medical Center (NL76835.018.21, 4 November 2021). Informed consent was obtained from all subjects involved in the study in accordance with national guidelines.

### 2.2. Treatment and Clinical Assessments

CAR-T care was provided according to the summary of product characteristics of axi-cel, national and institutional guidelines. Any anti-cancer treatment provided between leukapheresis and lymphodepleting chemotherapy (fludarabine and cyclophosphamide) was defined as bridging therapy (BT). Cytokine release syndrome (CRS) and immune effector cell-associated neurotoxicity syndrome (ICANS) were graded using the American Society for Transplantation and Cellular Therapy (ASTCT) criteria [18]. Cytopenias were graded using the Common Terminology Criteria for Adverse Events (CTCAE). CD4 and CD8 depletion was defined as a count <0.2 × 10^9^/L, hypogammaglobulinemia as IgG < 6 g/L and B-cell aplasia as B-cells/lymphocytes < 0.5%. Infections were diagnosed and managed according to national and institutional guidelines [19]. We collected information on the type (site/pathogen) and timing (within the first month after CAR-T infusion, between Month 1 and Month 12 after CAR-T infusion and more than 1 year after CAR-T infusion) of any type and any grade infections occurring after CAR-T infusion. Immunoglobulin was substituted intravenously in cases of serious or recurrent infections and IgG < 4 g/L [20]. The treatment response was assessed according to the Lugano classification [21]. The overall response rate (ORR) was defined as the percentage of patients achieving a partial metabolic response (PR) or complete metabolic response (CR) after CAR-T and the best ORR as the best achieved response at any time. Stable disease (SD) and progressive disease (PD) were regarded as no response.

### 2.3. Statistical Analyses

Descriptive statistics were used to analyze the clinical characteristics. The Kaplan–Meier method was used to analyze the overall survival (OS) and progression-free survival (PFS). Survival was defined as the time from CAR-T infusion until death from any cause (OS) or until progression or death (PFS), whichever came first and in case no event occurred, patients were censored at last follow-up. Cox proportional hazards regression models were used for univariable and multivariable analysis for OS and PFS. Variables with at least marginal association with PFS/OS from the univariable analysis (*p* < 0.1) were included in the multivariable model. The results are expressed as a hazard ratio (HR) with a 95% confidence interval (CI). A *p*-value of ≤0.05 was considered statistically significant. Progression and non-relapse mortality (NRM) cumulative incidence rates were analyzed by the method of Fine and Gray, with death in remission and progression counted as competing risks, respectively. HR-QoL data were evaluable if baseline and at least one follow-up assessment was available. In case of disease progression, only HR-QoL data until progression were included for analyses. Summary statistics were calculated for the EORTC QLQ-C30/EQ-5D-5L total and change scores. A change score above 0 indicates a QoL improvement with a change score ≥7 (EQ-5D-5L VAS) or ≥10 (EORTC QLQ-C30) considered as a clinically meaningful change [22,23]. Statistical analyses were performed using R software (Version 4.2.1).

## 3. Results

### 3.1. Patient Selection and Characteristics

**Figure 1:**
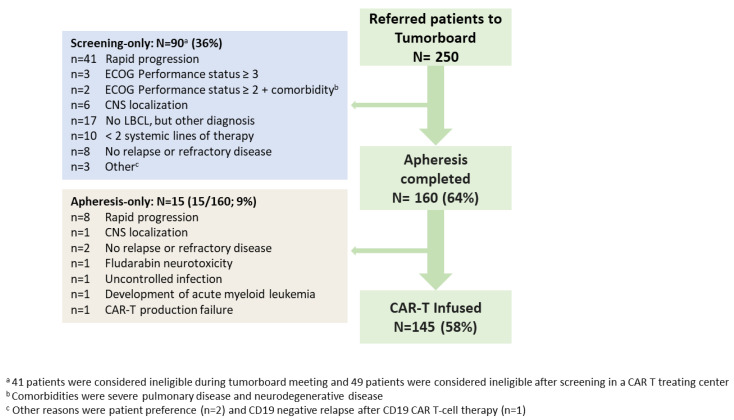
Flowchart of national patient selection for commercially available CD19-directed CAR-T therapy: all patients referred to the Dutch CAR-T tumorboard between May 2020 until May 2022 and reasons for ineligibility. Abbreviations: CNS: central nervous system, ECOG: Eastern Cooperative Oncology Group, LBCL: large B-cell lymphoma.

Between May 2020–May 2022, 250 patients were referred to the tumorboard for treatment with axi-cel; in total, 64% (n = 160) underwent leukapheresis, of whom 145 patients received an axi-cel infusion (apheresis-only: 15/160; 9%) (Figure 1). Of the 105 patients (screening-only: n = 90; apheresis-only: n = 15) who did not receive an axi-cel infusion, 40 patients (screening-only: n = 38; apheresis-only: n = 2) were ineligible because they did not meet the axi-cel product registration label (other diagnosis than LBCL, no (histological) proven R/R disease, <2 prior lines of systemic therapy, no wish to be treated with CAR-T, or R/R disease after prior CD19-CAR-T). These 40 patients were excluded from further analyses. Of the 210 included patients with the appropriate indication for axi-cel treatment, 65 patients (screening-only: n = 52; apheresis-only: n = 13) did not receive axi-cel, with rapid progression as the main reason (49/65; 75%).

**Figure 2:**
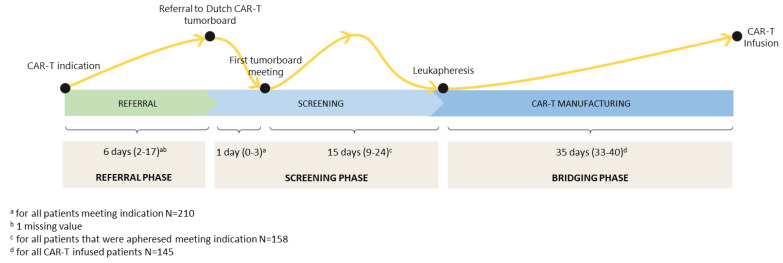
Time path from indication for CD19-directed CAR-T therapy to CAR-T infusion (median duration in days (IQR)).

The median time between CAR-T indication (based on date PET-CT scan) and referral to the tumorboard was 6 days (interquartile range (IQR): 2–17), and the median time between referral and discussion in the tumorboard was 1 day (IQR: 0–3) (Figure 2). The median time between the tumorboard meeting and leukapheresis was 15 days (IQR: 9–24), and the median time between leukapheresis and CAR-T infusion was 35 days (IQR: 33–40). For infused patients, the median time between referral and CAR-T infusion was 54 days (IQR: 45–68).

The baseline characteristics of the 145 infused patients at screening for CAR-T are presented in Table 1. The median age was 60 years (range: 21–84), 34% of patients were female and most patients were diagnosed with DLBCL (50%) followed by tFL (33%), HGBCL (14%) and PMBCL (3%). The baseline characteristics of all 210 patients with the appropriate indication as per the axi-cel product registration label are presented in Table S1 for the total cohort and for the three subgroups (i.e., screening-only, apheresis-only and infused).

**Figure 3:**
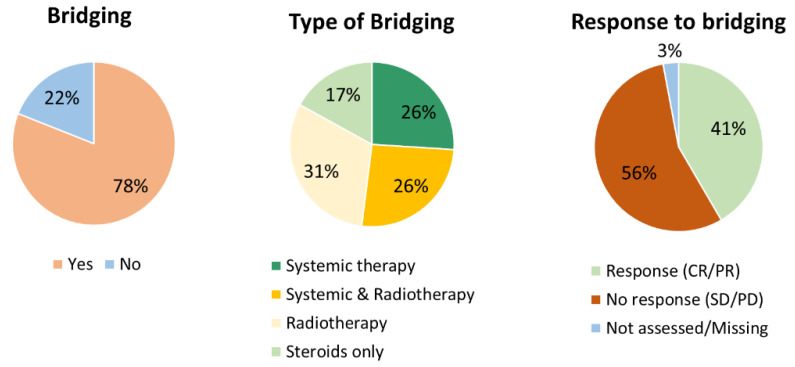
Bridging therapy used in CAR-T infused patients. Abbreviations: CR: complete response, PD: progressive disease, PR: partial response, SD: stable disease.

In total, 78% (n = 113) of the CAR-T infused patients received BT (Figure 3). Of these 113 patients, 26% (n = 30) received systemic therapy, 31% (n = 35) received radiotherapy, 26% (n = 29) received a combination of systemic therapy and radiotherapy and 17% (n = 19) received high-dose steroids only. In 110/113 patients receiving BT, the response was assessed and 47 patients (41%) responded to BT. The types of systemic therapy used for bridging and response to bridging are provided in Table S2. In addition to high-dose steroids, the next most frequently used systemic therapy regimen was rituximab/polatuzumab/bendamustine. The systemic therapy regimens with the highest response rates were rituximab/ifosfamide/gemcitabine/vinolrebine/prednisone (2/3: 67%); high-dose cytarabine (1/2: 50%), although used in a very low frequency; and rituximab/polatuzumab/bendamustine (7/16: 44%).

### 3.2. Efficacy

**Figure 4:**
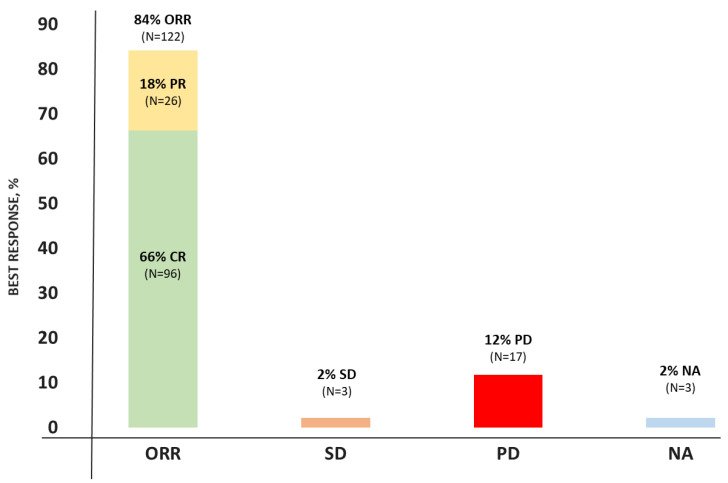
Best overall response rates to CAR-T. Abbreviations: CR: complete response, NA: not available, ORR: overall response rate, PD: progressive disease, PR: partial response, SD: stable disease.

**Figure 5:**
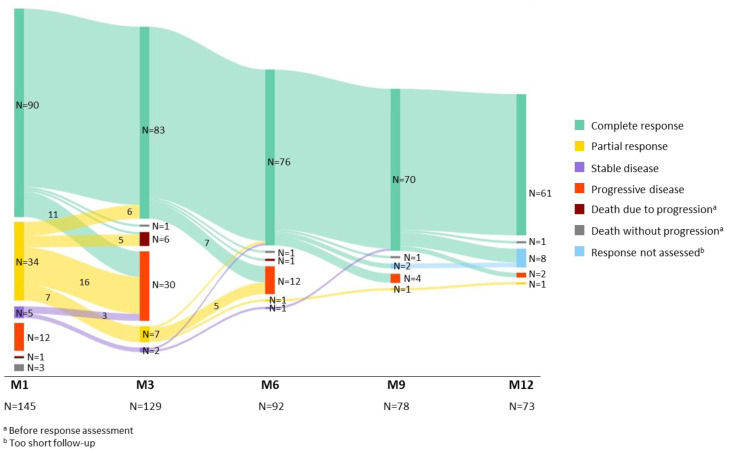
Response to axicabtagene ciloleucel over time in the first year post-infusion until progression or death.

The response was assessed in 142/145 infused patients (3 patients died due to other reasons than disease progression before response assessment). The best ORR was 84% with 66% of patients achieving CR (Figure 4). The median time to response was 1 month (min–max: 1–9). Figure 5 shows the conversion of the treatment response over time in the first year post-infusion, until progression or death (whichever came first). In total, seven patients converted from a PR to a CR: six patients at Month 3 and one patient at Month 6. Additionally, two patients with SD at Month 1 converted to a CR, one patient at Month 6 and one patient at Month 9. The proportion of patients with a PR or CR and subsequent relapse declined over time.

**Figure 6:**
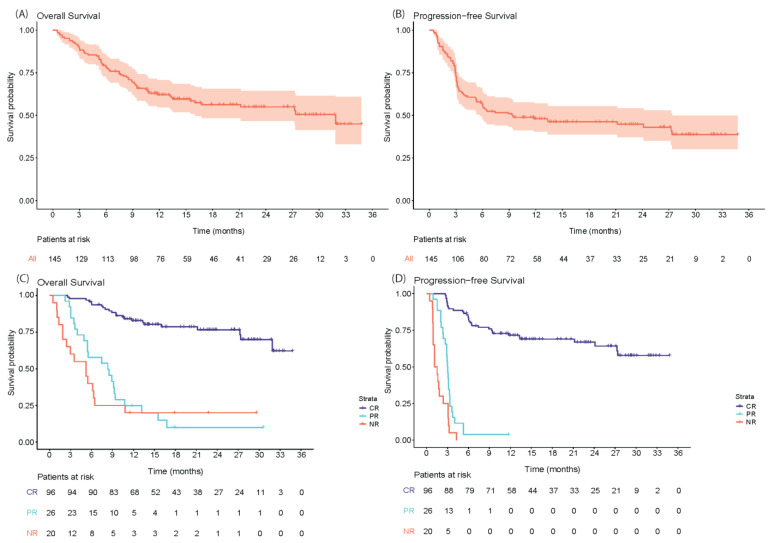
Overall survival and progression-free survival from CAR-T infusion for all CAR-T infused patients (**A**,**B**) and stratified per best response (**C**,**D**). Abbreviations: CR: complete response, NR: no response, PR: partial response.

The OS and PFS from CAR-T infusion for the total cohort and stratified per best ORR are shown in Figure 6. At a median follow-up of 13.0 months (IQR: 7.0–22.6; median follow-up of patients alive: 19.2 months (IQR: 13.6–27.8)), the median OS for the total cohort was 31.9 months (95% CI: 16.0—not reached (NR)) with a 12-month OS-rate of 62.2% (95% CI: 54.7–70.7). The median PFS was 9.4 months (95% CI: 5.8–27.4) with a 12-month PFS-rate of 48.0% (95% CI: 40.5–56.9%). As 3 patients died before response assessment, the survival stratified per best ORR could only be assessed for 142 patients. The median OS and PFS for patients with CR as the best ORR were not reached (95% CI: 31.9-NR and 27.4-NR, respectively). For patients with PR as the best ORR, the median OS and PFS were 8.3 months (95% CI: 5.4–10.8) and 3.0 months (95% CI: 2.7–3.4), respectively. The median OS and PFS for patients with SD/PD as the best ORR (i.e., non-responding patients) were 5.2 months (95% CI: 2.4–10.8) and 1.3 months (95% CI: 1.0–3.1), respectively. The OS and PFS from CAR-T infusion according to three subgroups based on BT (no bridging, response to bridging (CR/PR) and no response to bridging (SD/PD)) are provided in Figure S1. The median OS and PFS for patients who did not receive BT was not reached. For patients receiving BT and responding, the median OS and PFS was 27.4 months and 11.8 months, respectively, and for patients not responding, the median OS was 13.2 months and the median PFS was 3.1 months.

**Figure 7:**
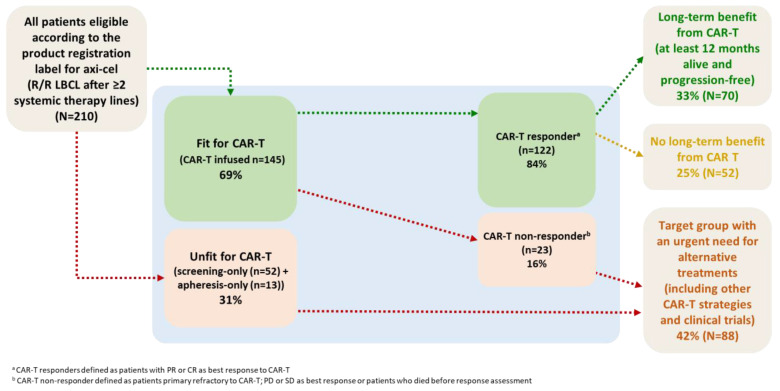
Flowchart describing the proportion of referred R/R LBCL patients with the appropriate indication as per the product registration label who benefit from axicabtagene ciloleucel. Abbreviations: Axi-cel: axicabtagene ciloleucel, LBCL: large B-cell lymphoma, R/R: relapsed/refractory.

The proportion of patients who benefit from CAR-T results from the combined effect of patient selection and the efficacy of axi-cel. In Figure 7, we describe this proportion among referred patients with an appropriate indication according to the axi-cel product registration label in the Netherlands. In this population, 33% had a long-term benefit from axi-cel, defined as being alive and progression-free at 1 year post-infusion, and 25% did initially respond to axi-cel but relapsed within 1 year. The other 42% of patients were unfit for axi-cel or not responding to axi-cel and in urgent need of alternative treatments (including clinical trials and alternative CAR-T strategies such as a shorter vein-to-vein time). 

Univariable analyses for OS (presented in Table S3) revealed significant associations for male sex and at screening for: elevated LDH (≥2xULN), IPI score ≥ 3 and no CR to first-line treatment (primary refractory disease), and at the time of infusion for: LDH (1 − 2xULN & ≥2xULN), performance status ≥2, platelets <75 × 10^9^/L, CRP > 50 mg/L, ferritin > 1000 µg/L, hemoglobin <6 mmol/L and no BT versus BT and no response to BT. In multivariable analysis for OS (Table 2A), an IPI score ≥ 3 at screening, LDH ≥ 2xULN and hemoglobin < 6 mmol/L at the time of infusion were associated with significant inferior OS. Univariable analyses for PFS (presented in Table S4) revealed significant associations for LDH at screening (LDH 2xULN) and infusion (1 − 2xULN & LDH ≥ 2xULN), platelets < 75 × 10^9^/L at time of infusion, CRP > 50 mg/L at the time of screening, ferritine > 1000 µg/L at the time of infusion and no BT versus BT and no response to BT. In multivariable analysis for PFS (Table 2B), no response to BT, an IPI score ≥ 3 at screening and hemoglobin < 6 mmol/L at the time of infusion were associated with a significant inferior PFS. 

### 3.3. Toxicity, Mortality and Hospital Admission

The median hospital admission duration (CAR-T infusion—discharge) was 14 days (min–max: 7–78), with 14% of patients admitted to the intensive care unit (Table 3). The majority of patients experienced any grade CRS (92%; grade ≥3: 5%) and 62% of patients experienced ICANS (grade ≥3: 22%). Within the first month after CAR-T, 26% of patients experienced an infection (any grade), 43% of patients experienced an infection between Month 1 and Month 12 and 43% of patients still experienced an infection more than one year after CAR-T infusion. At Month 3, 15% of patients had an ongoing grade ≥3 thrombocytopenia, 7% a grade ≥3 anemia and 34% a grade ≥3 neutropenia. One year after CAR-T, at least 49% of patients still had CD4 depletion, 33% CD8 depletion, 53% B-cell aplasia and 68% hypogammaglobulinemia. Between discharge and the first month post-CAR-T infusion, 18 patients (12%) were seen at the emergency room (ER) or were readmitted, of whom 7/18 patients were readmitted due to CAR-T related toxicity (mainly neurotoxicity). Between Months 1 and 3 post-CAR-T, 26 patients were seen at the ER and 19/26 patients were readmitted due to CAR-T related toxicity (mainly infections). Of the 64 patients who died (all causes of death are described in Table S5), 48 patients (75%) died due to progressive disease, 11 patients (8%) died when they were still in complete remission or before the first response assessment, due to an infection (n = 6), ICANS (n = 2) or AML/MDS (n = 3). The cumulative incidence of NRM at 12 months was 5% (Figure S2).

### 3.4. Health-Related Quality of Life

**Figure 8:**
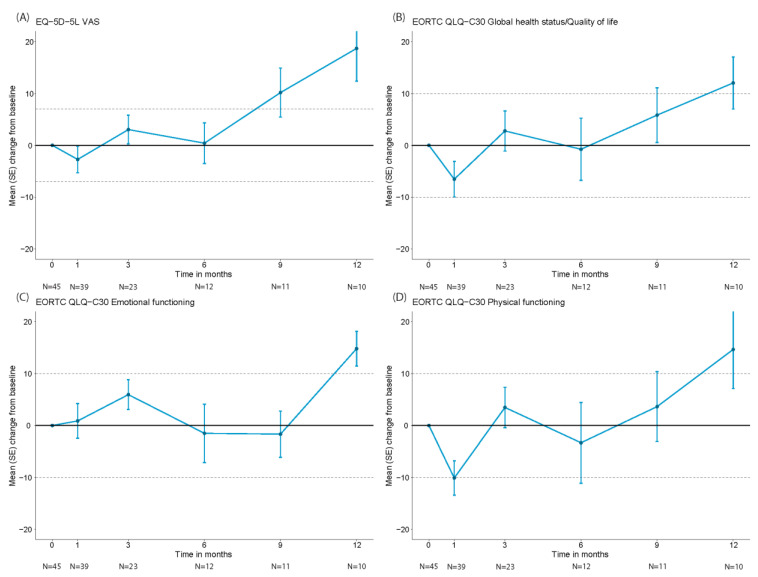
Mean changes from baseline in HR-QoL domain scores over time for EQ-5D-5L VAS score (**A**), EORTC QLQ-C30 global health status/quality of life (**B**), EORTC QLQ-C30 emotional functioning (**C**), EORTC QLQ-C30 physical functioning (**D**).

HR-QoL was assessed in a subset of 45 patients who all had a baseline and at least one follow-up assessment (baseline characteristics of these 45 patients are provided in Table S6). Data on HR-QoL at Months 1, 3, 6, 9 and 12 were available for 39, 23, 12, 11 and 10 patients, respectively. Reasons for missing HR-QoL data at follow-up included disease progression, death and logistical challenges. The mean scores at baseline for the EORTC QLQ-C30 global health status (GHS)/QoL physical and emotional functioning domains were 68.9 (standard deviation (SD): 17.7), 79.0 (SD: 23.7) and 74.6 (SD: 19.6), respectively. For the EQ-5D-5L VAS-score, the mean score at baseline was 68.4 (SD: 19.6). Initially, the mean change scores from baseline for the EQ-5D-5L overall health VAS and the EORTC QLQ-C30 GHS/QoL and physical functioning domains showed worsening scores at Month 1, which were clinically meaningful for the EORTC QLQ-C30 physical functioning domain (Figure 8). From Month 9 onwards the change scores for the EQ-5D-5L overall health VAS-score showed clinical meaningful improvement. Change scores for the EORTC QLQ-C30 GHS/QoL and physical functioning domains were clinically meaningfully improved at Month 12. The EORTC QLQ-C30 emotional functioning domain showed an initial non-clinical meaningful improvement until M3 and a slight worsening at Months 6 and 9 but a clinically meaningfully improvement at Month 12. The proportion of patients experiencing a clinically meaningful deterioration in overall health (measured with the EQ-5D-5L overall health VAS-score) was declining over time from 36% at Month 1 to 22%, 17%, 0% and 0% at Months 3, 6, 9 and 12, respectively (Figure S3). Additionally, the proportion of patients experiencing a clinically meaningful improvement in overall health was increasing from 26% at Month 1 to 30%, 33%, 36% and 60% at Months 3, 6, 9 and 12, respectively. 

## 4. Discussion

CAR-T therapy is one of the most significant advances in the treatment landscape of R/R LBCL over the past decades and its application is expanding to earlier lines [24,25,26,27,28,29]. Real-world results have demonstrated that CD19-CAR-T therapy can be applied to a more diverse patient population, but outcomes substantially differ across countries. Recently, some potential explanations for inferior survival for patients treated in Europe compared to the US were provided. High-risk disease and a longer vein-to-vein time were identified as main contributors to poor survival, highlighting the importance of patient selection and optimal logistics [15].

In the Netherlands, we have established a unique infrastructure for nationwide referral, eligibility assessment and data collection facilitated by a tumorboard, comprising CAR-T experts from all CAR-T treatment centers. This infrastructure not only provides equal access, expert-directed patient selection and high-quality care, but also allows for the evaluation of the outcomes of a consecutive population-based cohort of all referred R/R LBCL patients. The aims of this study were to evaluate the real-world outcomes of CD19-CAR-T in the Dutch population, to transparently report on all important aspects of the treatment trajectory, to show the underreported effect of CAR-T on HR-QoL and to identify the proportion of referred R/R LBCL patients ineligible for CAR-T for whom there is still an unmet medical need. In contrast to other European cohorts that evaluated the real-world outcomes of both axi-cel and tisa-cel, we only evaluated axi-cel for adult R/R LBCL patients after ≥2 systemic therapy lines, as it was the only product and indication reimbursed until May 2022 in the Netherlands. Although the reported time intervals between CAR-T indication, referral and evaluation in the Dutch tumorboard were short, due to logistics and manufacturing time it still takes a median of 53 days to receive CAR-T infusion after tumorboard approval. This is comparable to some but shorter than other European cohorts (UK: 56 days, GLA/DRST: 66 days) [11,12]. The outcomes in our population of axi-cel infused patients are better or at least comparable to other studies. The best ORR in our cohort of 84% with a CR-rate (CRR) of 66% and a 12-month PFS- and OS-rate of 48% and 62%, respectively, is at least equal to ZUMA-1, the US-lymphoma consortium, the CIBMTR cohort and the French cohort DESCAR-TES and more favorable than the results from the UK-cohort, the GETH/Geltamo-cohort and the GLA/DRST-cohort (see results from these different cohorts in Supplementary S2) [5,8,10,11,12,13,14]. The efficacy of CAR-T therapy is not only influenced by product characteristics but also by patient selection. The proportion of the total referred R/R LBCL patient population with the appropriate indication as per the registration label who were treated and had a long term benefit from axi-cel was 33%. For a considerable proportion of patients referred for CAR-T there is thus still an urgent need for alternative treatments. The main reason patients with the appropriate indication were not selected was rapidly progressive disease (N = 49/210, 23%), based on expert opinion, in the absence of clearly defined criteria. This was most often decided before leukapheresis, during screening (41/49, 83%), contributing to a low percentage of patients apheresed and not proceeding to infusion. In our axi-cel-infused population, we identified low hemoglobin (<6 mmol/L) and a high IPI score (≥3) to be significantly associated with both inferior OS and PFS and, in addition, an elevated LDH (≥2x ULN) for OS and no response to bridging for PFS. It is plausible that these characteristics are surrogate markers for a more aggressive disease biology. However, to fully understand which factors determine and predict the outcome and can be used in upfront decision making, an in-depth analysis of all contributors (i.e., CAR-T product specifications, disease biology characteristics, radiomics) and their interactions in large datasets of multiple (national) cohorts are needed.

As the outcomes of axi-cel might be influenced by BT strategies and the response to BT, we also evaluated BT in our cohort. Previously, it has been reported that rituximab/polatuzumab/bendamustine and radiotherapy are associated with the highest response rates to BT [30]. These BT strategies were also most frequently used in our cohort. Whether BT aiming at reduction in tumor bulk or no BT is better in patients with less aggressive disease remains a point of discussion and can only definitely be answered by conducting a randomized trial; a more pragmatic approach could be a trial within cohorts (TwiCs) design study in a real-world setting [31]. The incidence of any grade (62%) and grade ≥3 ICANS (22%) was somewhat higher in our cohort compared to other European cohorts (while lower than in ZUMA-1), but we found a slightly lower incidence of grade ≥3 CRS (5%) and a lower 12-month NRM of 5% [5,11,12,13,14]. Similar to other real-world cohorts, tocilizumab (71%) and steroids (64%) were frequently used for toxicity management and early/pre-emptive use has increased compared to the pivotal cohorts in ZUMA-1 [5,11,12,13,14]. We observed that three patients in remission died due to myelodysplastic syndrome (MDS)/acute myeloid leukemia (AML), underscoring the importance of adequate surveillance for MDS/AML after CAR-T. If post-CAR-T MDS/AML can be attributed to the CAR-T treatment itself, to the lymphodepleting chemotherapy or for example is merely a consequence of the cumulative exposure to prior DNA-damaging therapy still needs to be elucidated [32,33]. In line with other real-world cohorts, the main cause of NRM was infection. In fact, infection as a result of prolonged impairment of immune reconstitution is one of the major threats for CAR-T patients [34]. The risk of infection also leads to more fear and anxiety, especially during pandemics such as the SARS-CoV-2 pandemic [35]. Optimizing strategies to reduce susceptibility to infections is not only important to reduce the incidence of (severe) infections and NRM but also to improve HR-QoL [36]. In addition to clinical outcomes, we evaluated the effect of axi-cel on HR-QoL. A clinically meaningful worsening was seen in physical functioning 1 month post-infusion, possibly caused by acute toxicities. Clinically meaningful improvements were seen from Month 9 onwards in overall health and at Month 12 in GHS/QoL, emotional functioning and physical functioning. Although this is one of the first and largest real-world studies reporting on both clinical and HR-QoL outcomes in R/R LBCL patients treated with axi-cel in ≥3rd-line, our cohort is still too small to analyze associations between baseline characteristics, toxicities and HR-QoL. Due to logistical challenges HR-QoL data were collected in only a subset of patients (three CAR-T centers participated in the HR-QoL data collection) and data were only evaluated until progression. The analysis of this subset of patients might not accurately represent the effect of axi-cel on HR-QoL for the entire CAR-T-infused population, which is a limitation of this study. Electronic collection of HR-QoL data might help overcome the logistical challenges and can improve the completeness of HR-QoL data collection, including data after progression. However, HR-QoL in patients who have disease progression or relapse may be heavily influenced by subsequent therapies. In addition, as acute CAR-T-mediated-toxicities such as ICANS and CRS occur within the first month, earlier assessments of HR-QoL are needed to better evaluate the impact of these toxicities on HR-QoL. Tailored HR-QoL questionnaires, including patient-reported outcome (PRO) items for CAR-T specific symptoms, can be used to capture the experience of patients treated with CAR-T, including during the first weeks of treatment [37]. Additionally, PROs, collected by a core signs and symptoms checklist for acute CAR-T toxicity, can be used to facilitate outpatient treatment [38]. Our study provides important information on all key aspects of the CAR-T treatment trajectory, including population estimates and HR-QoL data, which can be used for budget-impact and cost-effectiveness analyses. To further optimize CAR-T treatment, matched comparisons between cohorts are needed to better understand the differences in outcomes across countries and to select best practices. Patient selection can play an important role in applying CAR-T in an effective and sustainable manner, but more research on predicting outcomes is needed to provide more guidance. For a substantial proportion of patients, there is still a high unmet need for novel therapies.

## 5. Conclusions

This is one of the first real-world population-based studies transparently reporting on all important aspects of the CAR-T treatment trajectory from the time of CAR-T indication until infusion and beyond, evaluating both clinical and HR-QoL outcomes in R/R LBCL patients treated with axi-cel in ≥3rd-line. In the Netherlands, the outcomes of axi-cel are at least equal to the ZUMA-1 results and comparable to or more favorable than other European real-world cohorts. Clinically meaningful improvements in several HR-QoL domains were observed from Month 9 post-infusion onwards. Large and complete real-world datasets are needed to better predict which patients benefit from CAR-T and which patients should be treated with alternative therapies to further optimize patient selection.

## Figures and Tables

**Table 1:** Baseline characteristics at screening for CAR-T infused patients.

	CAR-T Infused Patients Cohort (N = 145)
**Median age (min–max)**	**60 years (21–84)**
**Female**, n (%)	50 (34%)
**Histological subtype**, n (%)	
DLBCL	73 (50%)
HGBCL (NOS/DH/TH)	20 (14%)
PMBCL	4 (3%)
tFL	48 (33%)
**IPI score**, n (%)	
Low	28 (19%)
Low-intermediate	49 (34%)
High-intermediate	41 (28%)
High	13 (9%)
Incompletely assessed, n (%)	14 (10%)
**Stage III–IV**, n (%)	124 (86%)
**Bulky disease (nodal ≥ 10 cm and/or extranodal ≥ 5 cm)**, n (%)	50 (34%)
Missing	2 (1%)
**Extranodal sites present**, n (%)	98 (68%)
2 Sites	26 (18%)
≥3 Sites	16 (11%)
Unknown number of sites	2 (1%)
**ECOG performance status**, n (%)	
0	85 (59%)
1	49 (34%)
≥2	11 (8%)
**Primary refractory** ^**a**^, n (%)	88 (61%)
**Disease status**, n (%)	
Relapse after last therapy line	29 (20%)
Refractory to last therapy line	116 (80%)
**Prior therapy lines**, median (min–max)	2 (2–6)
≥3	38 (26%)
**Prior transplant**, n (%)	
Autologous SCT	42 (29%)
Allogeneic SCT	3 (2%)
**LDH**, n (%)	
>1x ULN–2x ULN	54 (37%)
≥2x ULN	22 (15%)
Missing	12 (8%)
**Hemoglobin < 6 mmol/L**, n (%)	44 (30%)
Missing	2 (1%)
**Ferritin > 1000 µg/L**, n (%)	20 (14%)
Missing	82 (57%)
**CRP > 50 mg/L**, n (%)	28 (19%)
Missing	43 (30%)
**Platelets < 75 × 10^9^/L**, n (%)	17 (12%)
Missing	3 (2%)
**Neutrophil count < 0.5 × 10^9^/L**, n (%)	7 (5%)
Missing	30 (21%)
**Lymphocyte count < 0.5 × 10^9^/L**, n (%)	27 (19%)
Missing	40 (28%)

^a^ Primary refractory was defined as no complete response to first-line treatment. Abbreviations: DLBCL: diffuse large B-cell lymphoma, ECOG: Eastern Cooperative Oncology Group, HGBCL: high-grade B-cell lymphoma, IPI: international prognostic index, PMBCL: primary mediastinal large B-cell lymphoma, SCT: stem cell transplantation, tFL: transformed follicular lymphoma.

**Table 2:** Multivariable analysis for OS (**A**) and PFS (**B**).

Factor	HR (95% CI)	*p*-Value
(**A**). **Multivariable Analysis for OS**		
LDH elevated ≥ 2x ULN at time of infusion	7.40 (1.56–35.00)	0.0116 *
No response to bridging therapy	3.73 (0.75–18.45)	0.1072
Hemoglobin < 6 mmol/L at time of infusion	3.18 (1.16–8.75)	0.0247 *
LDH elevated 1 − 2x ULN at time of infusion	2.68 (0.98–7.36)	0.0553
Response to bridging therapy	2.35 (0.43–12.88)	0.3240
IPI score high intermediate and high risk (≥3)	2.35 (1.06–5.2)	0.0354 *
Male sex	2.33 (0.82–6.62)	0.1125
Platelets < 75 × 10^9^/L at time of infusion	1.92 (0.72–5.15)	0.1935
ECOG performance status ≥ 2 at time of infusion	1.44 (0.24–8.56)	0.6899
ECOG performance status = 1 at time of infusion	1.07 (0.47–2.47)	0.8661
No CR to first-line treatment (primary refractory disease)	1.07 (0.43–2.65)	0.8864
LDH elevated 1 − 2x ULN at screening	0.91 (0.37–2.24)	0.8290
CRP > 50 mg/L at time of infusion	0.82 (0.27–2.49)	0.7230
Ferritin > 1000 µg/L at time of infusion	0.53 (0.21–1.35)	0.1856
LDH elevated ≥ 2x ULN at screening	0.44 (0.10–1.85)	0.2627
(**B**). **Multivariable analysis for PFS**		
No response to bridging therapy	2.68 (1.00–7.17)	0.0497 *
LDH elevated ≥ 2x ULN at time of infusion	2.31 (0.71–7.53)	0.1630
IPI score high intermediate and high risk (≥3)	2.28 (1.20–4.33)	0.0123 *
Hemoglobin < 6 mmol/L at time of infusion	2.27 (1.04–4.95)	0.0392 *
LDH elevated 1 − 2x ULN at time of infusion	1.75 (0.87–3.55)	0.1185
Response to bridging therapy	1.24 (0.44–3.49)	0.6876
Platelets < 75 × 10^9^/L at time of infusion	1.09 (0.52–2.28)	0.8101
CRP > 50 mg/L at time of infusion	1.06 (0.51–2.19)	0.8756
Ferritin > 1000 µg/L at time of infusion	0.79 (0.40–1.56)	0.4957
LDH elevated 1 − 2x ULN at screening	0.73 (0.36–1.49)	0.3861
LDH elevated ≥ 2x ULN at screening	0.64 (0.21–1.89)	0.4160

Abbreviations: CI: confidence interval, CR: complete response, ECOG: Eastern Cooperative Oncology Group, HR: hazard ratio, IPI: international prognostic index, OS: overall survival, PFS: progression-free survival. * Significant association at a *p*-value of ≤0.05 considered statistically significant.

**Table 3:** Toxicity of CAR-T treatment.

Event	
**CRS**, any grade, n (%)	133 (92%)
Grade ≥3	7 (5%)
Median time to onset (min–max)	2 days (0–9)
Median duration	5 days (1–7)
**ICANS**, any grade, n (%)	90 (62%)
Grade ≥3	32 (22%)
Median time to onset (min–max)	6 days (1–28)
Median duration	5 days (1–28)
**Tocilizumab use**, n (%)	103 (71%)
**Anakinra use**, n (%)	3 (2%)
**Steroids use**, n (%)	93 (64%)
**Any grade and any type infections**, n (%)	
Month <1	38 (26%)
Months 1–12	61 (43%)
Months >12	33 (43%)
**Thrombocytopenia at Month 3**, n (%)	59 (45%)
Grade ≥3	19 (15%)
Missing	5 (4%)
**Anemia at Month 3**, n (%)	95 (73%)
Grade ≥3	9 (7%)
Missing	5 (4%)
**Neutropenia at Month 3**, n (%)	62 (47%)
Grade ≥3	45 (34%)
Missing	6 (5%)
**CD4 < 0.2 × 10^9^/L at 1 year**, n (%)	37 (49%)
Missing	27 (36%)
**CD8 < 0.2 × 10^9^/L at 1 year**, n (%)	25 (33%)
Missing	27 (36%)
**B-cell aplasia (B-cells/lymphocytes < 0.5%) at 1 year**, n (%)	40 (53%)
Missing	26 (34%)
**Hypogammaglobulinemia (IgG < 6 g/L) at 1 year**, n (%)	52 (68%)
Missing	17 (22%)
**Median hospital admission duration** (min–max)	14 days (7–78)
**Patients that died during admission**, n (%)	4 (3%)
**ICU admission**, n (%)	20 (14%)
**Median ICU admission duration** (min–max)	3 days (1–19)
**ER visits and readmissions < Month 1**, n (%)	18 (12%)
Readmission for CAR-T related toxicity, n	7 (neurotoxicity 6, infection 1)
**ER visits and readmissions between Month 1–3**, n (%)	26 (18%)
Readmission for CAR-T related toxicity, n	19 (neurotoxicity 2, infection 17)

Abbreviations: CRS, cytokine release syndrome, ER: emergency room, ICANS: immune effector-cell associated neurotoxicity syndrome, ICU: intensive care unit.

## Data Availability

The data presented in this study are available from the corresponding author upon reasonable request.

## Supplementary Materials

The following supporting information can be downloaded at: https://www.mdpi.com/article/10.3390/cancers15174334/s1, Table S1. Baseline characteristics of all patients meeting the appropriate indication as per product registration label (total cohort), and for the three subgroups (i.e., screening-only, apheresis-only and infused). Table S2. The different types of systemic therapy used for bridging and response to bridging. Table S3. Univariable analysis for overall survival (OS). Table S4. Univariable analysis for progression-free survival. Table S5. Mortality with description of all causes of death. Table S6. Baseline characteristics of the subset of patients in which HR-QoL was assessed. Figure S1. Overall survival (A) and progression-free survival (B) from CAR-T infusion according to three subgroups based on bridging: no bridging, response to bridging (CR/PR) and no response to bridging (SD/PD). Figure S2. Non-relapse mortality (NRM) curve. Figure S3. Proportion of patients experiencing clinically meaningful deterioration or improvement in the EQ-5D-5L overall health VAS score over time. Supplementary S1. Dutch CAR-T Tumorboard eligibility criteria May 2020–May 2022. Supplementary S2. Efficacy outcomes of axi-cel in other real-world cohorts.
