# Supplementary material for: The Dutch CAR-T Tumorboard Experience: Population-Based Real-World Data on Patients with Relapsed or Refractory Large B-Cell Lymphoma Referred for CD19-Directed CAR T-Cell Therapy in The Netherlands
Source: Cancers (Basel). 2023 Aug 30;15(17):4334. doi: 10.3390/cancers15174334 (PMC10486925; doi:10.3390/cancers15174334)
Supplement: Supplementary file 1 [file cancers-15-04334-s001.zip › cancers-2513419-supplementary.pdf]

Supplementary Materials

The Dutch CAR-T Tumorboard Experience: Population-Based Real-World Data on Patients with Relapsed or Refractory Large B-Cell Lymphoma Referred for CD19-Directed CAR T-Cell Therapy in The Netherlands

Anne M. Spanjaart[1,2,3*] & Elise R. A. Pennings[1,2,3,4*], Pim G.N.J. Mutsaers[5], Suzanne van Dorp[6], Margot Jak[7], Jaap A. van Doesum[8], Janneke W. de Boer[8], Anne G.H. Niezink[9], Milan Kos[2,10], Joost S.P. Vermaat[11], Aniko Sijs-Szabo[11], Marjolein W.M. van der Poel[12], Inger S. Nijhof[2,13,14], Maria T. Kuipers[2,13], Martine E.D. Chamuleau[2,13], Pieternella J. Lugtenburg[5], Jeanette K. Doorduijn[5], Yasmina I.M. Serroukh[5], Monique C. Minnema[7], Tom van Meerten[8], Marie José Kersten[1,2,3] on behalf of the Dutch CAR-T tumorboard consortium

** These authors contributed equally to this work.*

Table S1. Baseline characteristics of all patients meeting the appropriate indication as per product registration label (total cohort), and for the three subgroups (i.e. screening-only, apheresis-only and infused)

	**Indication cohort N=210**	**Screening-only cohort N=52**	**Apheresis-only cohort N=13**	**Infused cohort N=145**
**Median age** (min-max)	62 years (21-84)	64 years (32-78)	64 years (41-77)	60 years (21-84)
**Female,** n (%)	73 (35%)	14 (27%)	9 (69%)	50 (34%)
**Histological subtype,** n (%)				
DLBCL	109 (52%)	29 (56%)	7 (54%)	73 (50%)
HGBCL	28 (13%)	5 (10%)	3 (23%)	20 (14%)
PMBCL	4 (2%)	0 (0%)	0 (0%)	4 (3%)
tFL	69 (33%)	18 (35%)	3 (23%)	48 (33%)
**IPI score,** n (%)				
Low	31 (15%)	3 (6%)	0 (0%)	28 (19%)
Low-intermediate	60 (29%)	8 (15%)	3 (23%)	49 (34%)
High-intermediate	59 (28%)	12 (23%)	6 (46%)	41 (28%)
High	34 (16%)	18 (35%)	3 (23%)	13 (9%)
Incompletely assessed	26 (12%)	11 (21%)	1 (8%)	14 (10%)
**Stage III/IV,** n (%)	187 (89%)	50 (96%)	13 (100%)	124 (86%)
**Bulky disease**[a]**,** n (%)	85 (40%)	30 (58%)	5 (38%)	50 (34%)
Missing	4 (2%)	2 (4%)	0 (0%)	2 (1%)
**Extranodal site present,** n (%)	157 (75%)	47 (90%)	12 (92%)	98 (68%)
2 sites	43 (20%)	16 (31%)	1 (8%)	26 (18%)
≥ 3 Sites	33 (16%)	14 (27%)	3 (23%)	16 (11%)
Unknown number of sites	4 (2%)	2 (4%)	0 (0%)	2 (1%)
**ECOG-PS,** n (%)				
0	97 (46%)	10 (19%)	2 (15%)	85 (59%)
1	79 (38%)	21 (40%)	9 (69%)	49 (34%)
≥ 2	32 (15%)	19 (37%)	2 (15%)	11 (8%)
Missing	2 (1%)	2 (4%)	0 (0%)	0 (0%)
**Primary refractory**[b]**,** n (%)	131 (62%)	35 (67%)	8 (62%)	88 (61%)
**Disease status,** n (%)				
Relapse after last therapy line	40 (19%)	10 (19%)	1 (8%)	29 (20%)
Refractory to last therapy line	170 (81%)	42 (81%)	12 (92%)	116 (80%)
**Prior therapy lines,** median (min-max)	2 (2-6)	2 (2-5)	2 (2-3)	2(2-6)
≥ 3	54 (26%)	15 (29%)	1 (8%)	38 (26%)
**Prior transplant, n (%)**				
Autologous SCT	55 (26%)	11 (21%)	2 (15%)	42 (29%)
Allogenic SCT	3 (1%)	0 (0%)	0 (0%)	3 (2%)
**LDH,** n (%)				
> 1xULN – 2x ULN	78 (37%)	19 (37%)	5 (38%)	54 (37%)
≥ 2x ULN	42 (20%)	15 (29%)	5 (38%)	22 (15%)
Missing	21 (10%)	9 (17%)	0 (0%)	12 (8%)
**Hemoglobin < 6 mmol/L,** n (%)	68 (32%)	18 (35%)	6 (46%)	44 (30%)
Missing	16 (8%)	13 (25%)	1 (8%)	2 (1%)
**Ferritin > 1000 µg/L,** n (%)	33 (16%)	10 (19%)	3 (23%)	20 (14%)
Missing	117 (56%)	32 (62%)	3 (23%)	82 (57%)
**CRP > 50 mg/L,** n (%)	42 (20%)	13 (25%)	1 (8%)	28 (19%)
Missing	72 (34%)	26 (50%)	3 (23%)	43 (30%)
**Platelets <75 x10^9/L,** n (%)	29 (14%)	9 (17%)	3 (23%)	17 (12%)

Missing	14 (7%)	11 (21%)	0 (0%)	3 (2%)
**Neutrophil count <0.5 x10⁹/L,** n (%)	10 (5%)	3 (6%)	0 (0%)	7 (5%)
Missing	44 (21%)	13 (25%)	1 (8%)	30 (21%)
**Lymphocyte count <0.5 x10⁹/L,** n (%)	45 (21%)	12 (23%)	6 (46%)	27 (19%)
Missing	56 (27%)	16 (31%)	0 (0%)	40 (28%)

[a] Bulky disease: nodal ≥10 cm and/or extranodal ≥ 5 cm,

[b] Primary refractory: no complete response to first-line treatment

Abbreviations: DLBCL: diffuse large B-cell lymphoma, ECOG: Eastern Cooperative Oncology Group, HGBCL: high-grade B-cell lymphoma, IPI: international prognostic index, PMBCL: primary mediastinal large B-cell lymphoma, SCT: stem cell transplantation, tFL: transformed follicular lymphoma

Table S2. The different types of systemic therapy used for bridging and response to bridging

**Regimen**	**Patients, N**	**Patients with response (CR/PR) to bridging, N** (%)
R Gemcitabine/Oxaliplatin	6	2 (33%)
R Polatuzumab/Bendamustine	16	7 (44%)
R (Ifosfamide)/GEV	3	2 (67%)
R VIM	2	0 (0%)
R GDP	5	1 (20%)
Brentuximab	1	0 (0%)
(R) MPNS/Dexamethasone	35	8 (23%)*
HD AraC	2	1 (50%)
HD MtX	1	0 (0%)
R CHOP	3	1 (33%)
R CEOP	1	0 (0%)
R Lenalidomide	2	0 (0%)

Abbreviations: CHOP: cyclophosphamide hydroxydaunorubicin, vincristine, prednisolone, CEOP: cyclophosphamide etoposide vincristine prednisolone, GDP: gemcitabine, dexamethasone, cisplatin, GEV: gemcitabine vinolrebine prednisone, HD AraC:, high-dose cytarabine, HD MtX: high-dose methotrexate, MPNS: methylprednisolone, R: Rituximab, VIM: etoposide, ifosfamide, mixantrone

*For 2 patients response assessment was not available.

Table S3. Univariable analysis for overall survival (OS)

**Factor**	**Events/N**	**HR (95% CI)**	**P-Value**
**Age**			
< 65 years	42/97	1.00	0.834
≥ 65 years	22/48	1.057 (0.63-1.77)	
**Sex**			
Female	17/50	1.00	0.045
Male	47/95	1.767 (1.01-3.08)	
**ECOG at lymphodepleting chemotherapy**			
0	19/54	1.00	
1	32/67	1.66 (0.94-2.93)	0.083
≥2	7/13	2.71 (1.13-6.49)	0.025
**Disease Stage**			
I/II	7/21	1.00	0.208
III/IV	57/124	1.66 (0.76-3.63)	
**LDH at screening**			
LDH normal	18/57	1.00	
LDH > 1xULN – 2x ULN	23/54	1.67 (0.90-3.11)	0.105

LDH ≥ 2x ULN	17/22	4.40 (2.26(8.73)	0.0000156
**LDH at infusion**			
LDH normal	30/92	1.00	
LDH > 1xULN – 2x ULN	23/36	2.91 (1.69-5.03)	0.000126
LDH ≥ 2x ULN	10/12	5.73 (2.77-11.84)	0.00000249
**Relapsed or Refractory**			
Refractory	54/116	1.00	0.198
Relapse	10/29	0.64 (0.33-1.26)	
**IPI score**			
0-2	27/77	1.00	0.00282
3-5	30/54	2.22 (1.32-3.74)	
**Extranodal sites**			
0-2	56/129	1.00	0.194
≥ 3	8/16	1.64 (0.78-3.46)	
**Bulky disease**			
No	40/93	1.00	0.865
Yes	22/50	1.05 (0.62-1.76)	
**Primary refractory**			
No	19/57	1.00	0.0327
Yes	45/88	1.80 (1.05-3.07)	
**Platelets at infusion**			
≥ 75	43/113	1.00	0.000102
< 75	21/30	2.83 (1.68-4.80)	
**CRP at infusion**			
≤ 50	32/94	1.00	0.0000279
> 50	23/33	3.17 (1.85-5.44)	
**Ferritin at infusion**			
≤ 1000	20/56	1.00	0.00318
>1000	28/45	2.38 (1.34-4.23)	
**Hemoglobin at infusion**			
≥ 6	24/82	1.00	0.0000162
< 6	40/63	3.08 (1.85-5.14)	
**Bridging**			
No bridging	4/32	1.00	
Bridging with response	22/47	4.81 (1.66-13.98)	0.00386
Bridging with no response	37/63	6.88 (2.45-19.32)	0.000256
**Prior SCT**			
No	48/103	1.00	0.260
Yes	16/42	1.39 (0.41-1.27)	
**Prior therapy lines**			
0-2	50/107	1.00	0.364
>2	14/38	0.76 (0.42-1.38)	
**Time between indication and referral**			
> 7 days	29/66	1.00	0.955
≤ 7 days	35/79	0.99 (0.60-1.61)	
**Time between tumorboard meeting and apheresis**			
> 3 weeks	18/42	1.00	0.978
≤ 3 weeks	46/103	0.99 (0.58-1.71)	

Abbreviations: CI: confidence interval, ECOG: Eastern Cooperative Oncology Group, HR: hazard ratio, IPI: international prognostic index

Table S4. Univariable analysis for progression-free survival

Factor	Events/N	HR (95% CI)	P-Value
**Age**			
< 65 years	53/97	1.00	0.779
≥ 65 years	28/48	1.07 (0.68-1.69)	
**Sex**			
Female	25/50	1.00	0.272
Male	56/95	1.30 (0.81-2.09)	
**ECOG at infusion**			
0	29/54	1.00	
1	37/12	1.15 (0.70-1.87)	0.576
≥2	8/13	1.72 (0.79-3.76)	0.176
**Disease Stage**			
I/II	9/21 72/124	1.00	0.198
III/IV		1.58 (0.79-3.15)	
**LDH at screening**			
LDH normal	26/57	1.00	
LDH > 1xULN – 2x ULN	29/54	1.39 (0.82-2.36)	0.225
LDH ≥ 2x ULN	18/22	3.38 (1.84-6.21)	0.0000889
**LDH at infusion**			
LDH normal	45/92	1.00	
LDH > 1xULN – 2x ULN	24/36	1.88 (1.15-3.10)	0.000191
LDH ≥ 2x ULN	10/12	3.73 (1.87-7.46)	0.0126
**Relapsed or Refractory**			
Refractory	66/116	1.00	0.303
Relapse	15/26	0.74 (0.42-1.30)	
**IPI score**			
0-2	35/77 37/54	1.00	0.000964
3-5		2.18 (1.37-3.47)	
**Extranodal sites**			
0-2	71/129	1.00	0.284
≥ 3	10/16	1.44 (0.74-2.79)	
**Bulky disease**			
No	50/93	1.11 (0.70-1.76)	0.647
Yes	29/50		
**Primary refractory**			
No	29/57	1.00	0.149
Yes	52/88	1.40 (0.89-2.20)	
**Platelets at infusion**			
≥ 75	59/113	1.00	0.0137
< 75	22/30	1.86 (1.14-3.03)	
**CRP at infusion**			
≤ 50	48/94	1.00	0.00188
> 50	24/33	1.19 (1.36-3.60)	
**Ferritin at infusion**			
≤ 1000	28/56	1.00	0.0147
>1000	32/45	1.88 (1.133-3.13)	
**Hemoglobin at infusion**			
≥ 6	38/82	1.00	0.00255
< 6	43/63	1.96 (1.27-3.04)	

**Bridging**			
No bridging	9/32	1.00	
Bridging with response	27/47	2.48 (1.16-5.26)	0.018616
Bridging with no response	44/63	3.82 (1.86-7.84)	0.000257
**Prior SCT**			
No	61/103	1.00	0.39
Yes	20/42	0.82 (0.53-1.28)	
**Prior therapy lines**			
0-2	62/107	1.00	0.542
>2	19/28	0.85 (0.50-1.46)	
**Time between indication and referral**			
≤ 7 days	47/79	1.00	0.39
> 7 days	34/66	0.82 (0.53-1.28)	
**Time between tumorboard meeting and apheresis**			
≤ 3 weeks	59/103	1.00	0.787
> 3 weeks	22/42	0.93 (0.57-1.53)	

Abbreviations: CI: confidence interval, ECOG: Eastern Cooperative Oncology Group, HR: hazard ratio, IPI: international prognostic index

Table S5. Mortality with description of all causes of death

**Cumulative incidence of death**	64 (44%)
**1 year NRM**	5%
**Median time to death**	6.2 months (min-max: 0.5-31.9)
**Causes of death, n (%)**	64 (44%)
Progression	48
ICANS	2
Infection	9
Acute interstitial pneumonitis	1
CVA	1
AML/MDS	3
**Causes of death without progression (NRM), n (%)**	11 (8%)
ICANS	2
COVID-19	1
Pneumosepsis	1
Sepsis (invasive Aspergillosis)	1
Sepsis (E. coli)	1
Sepsis (Cellulitis)	1
Sepsis of unknown origin	1
AML from MDS	2
MDS with bone marrow failure	1
**Causes of death after progression, n (%)**	5 (3%)
Pneumosepsis	1
Acute interstitial pneumonitis	1
COVID-19	2
CVA	1

Abbreviations: AML/MDS: acute myeloid leukemia/myelodysplastic syndrome, CVA: cerebro vascular accident, ICANS: immune effector-cell associated neurotoxicity syndrome, NRM: non-relapse mortality

Table S6. Baseline characteristics of the subset of patients in which HR-QoL was assessed

	**HR-QoL subset N=45**
**Median age** (min-max)	58 years (28-79)
**Female,** n (%)	18 (40%)
**Histological subtype,** n (%)	
DLBCL	19 (42%)
HGBCL	7 (16%)
PMBCL	1 (2%)
tFL	18 (40%)
**IPI score,** n (%)	
Low	11 (24%)
Low-intermediate	14 (31%)
High-intermediate	17 (38%)
High	2 (4%)
Incompletely assessed	1 (2%)
**Stage III/IV,** n (%)	41 (91%)
**Bulky disease**[a]**,** n (%)	24 (53%)
Missing	0 (0%)
**Extranodal site present,** n (%)	33 (73%)
2 sites	12 (27%)
≥ 3 Sites	5 (11%)
Unknown number of sites	0 (0%)
**ECOG-PS,** n (%)	
0	38 (84%)
1	6 (13%)
≥ 2	1 (2%)
Missing	0 (0%)
**Primary refractory**[b]**,** n (%)	28 (62%)
**Disease status,** n (%)	
Relapse after last therapy line	11 (24%)
Refractory to last therapy line	34 (76%)
**Prior therapy lines,** median (min-max)	2 (2-5)
≥ 3	12 (27%)
**Prior transplant, n (%)**	
Autologous SCT	10 (22%)
Allogenic SCT	1 (2%)
**LDH,** n (%)	
> 1xULN – 2x ULN	15 (33%)
≥ 2x ULN	7 (16%)
Missing	1 (2%)

[a] Bulky disease: nodal ≥10 cm and/or extranodal ≥ 5 cm,

[b] Primary refractory: no complete response to first-line treatment

Abbreviations: DLBCL: diffuse large B-cell lymphoma, HGBCL: high-grade B-cell lymphoma, IPI: international prognostic index, PMBCL: primary mediastinal large B-cell lymphoma, tFL: transformed follicular lymphoma

Figure S1. Overall survival (A) and progression-free survival (B) from CAR-T infusion according to three subgroups based on bridging: no bridging, response to bridging (CR/PR) and no response to bridging (SD/PD)*

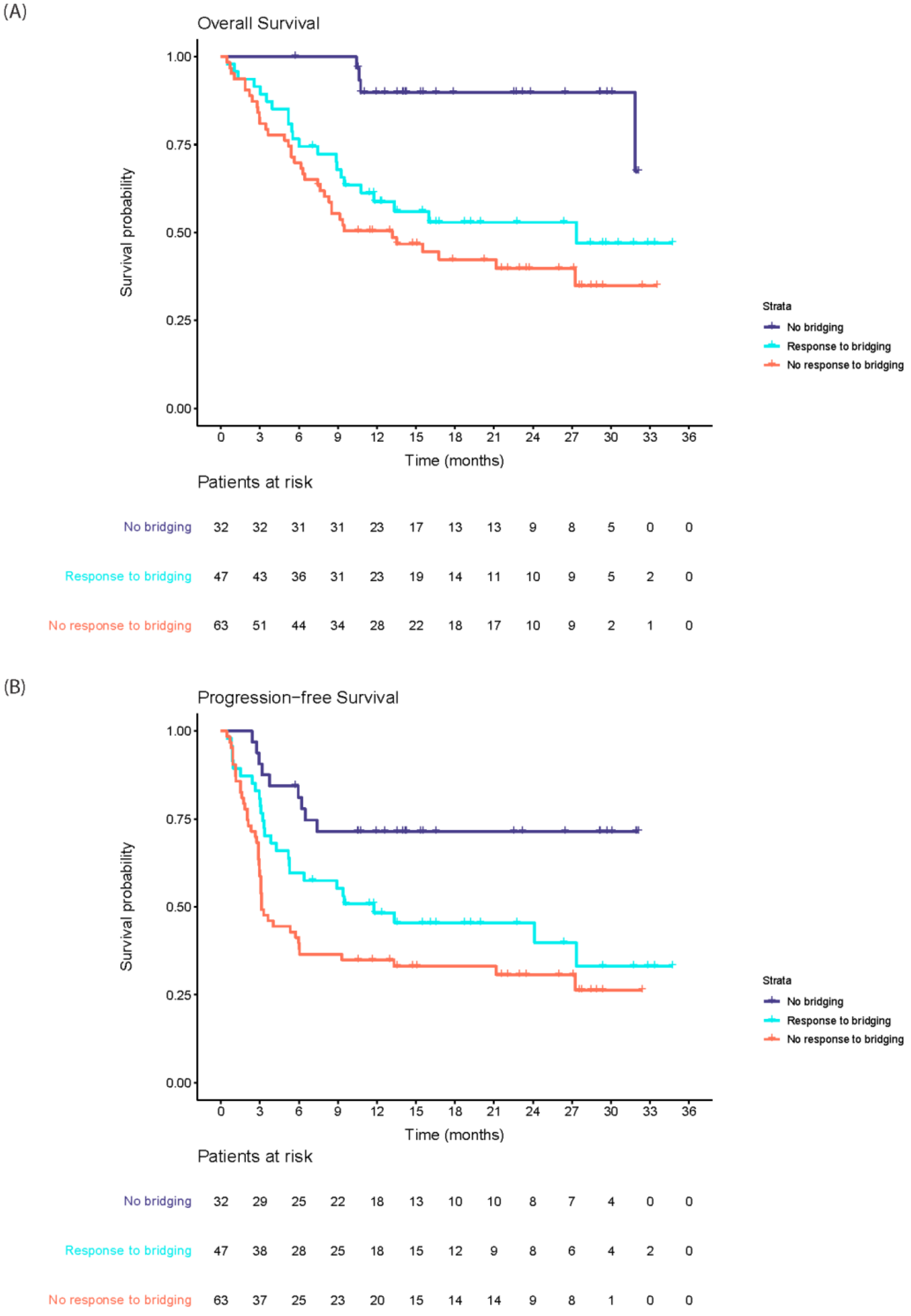

* As for 3 patients response to bridging therapy was not available, survival according to the three subgroups based on bridging could be assessed for 142 patients.

Figure S2. Non-relapse mortality (NRM) curve

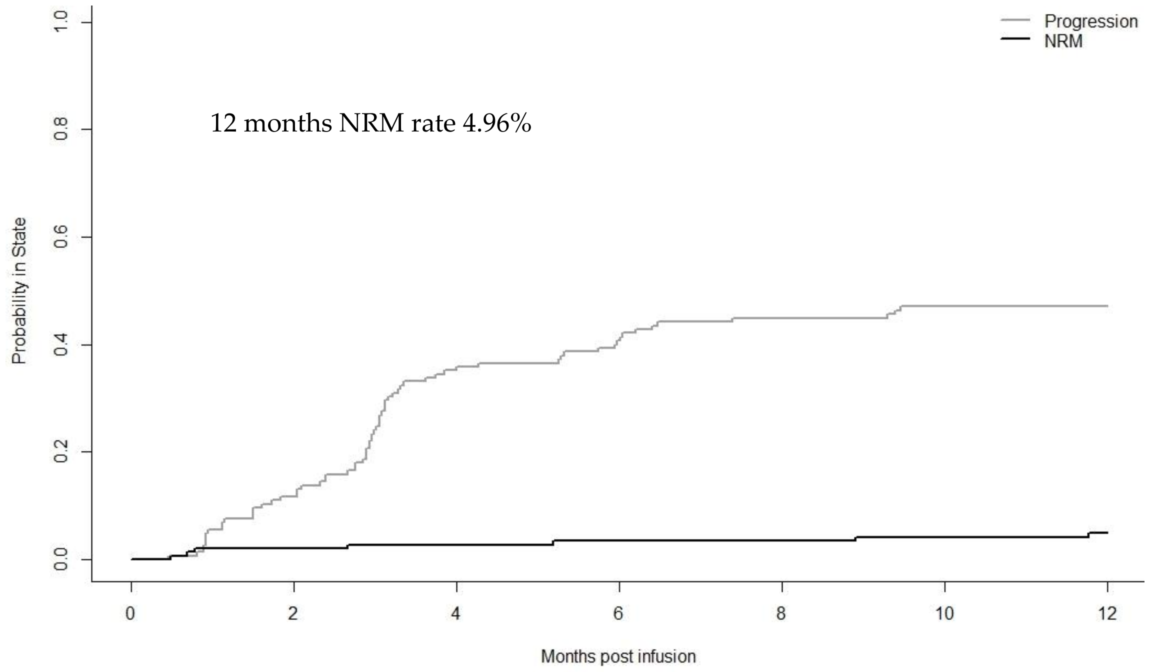

Figure S3. Proportion of patients experiencing clinically meaningful deterioration or improvement in the EQ-5D-5L overall health VAS score over time

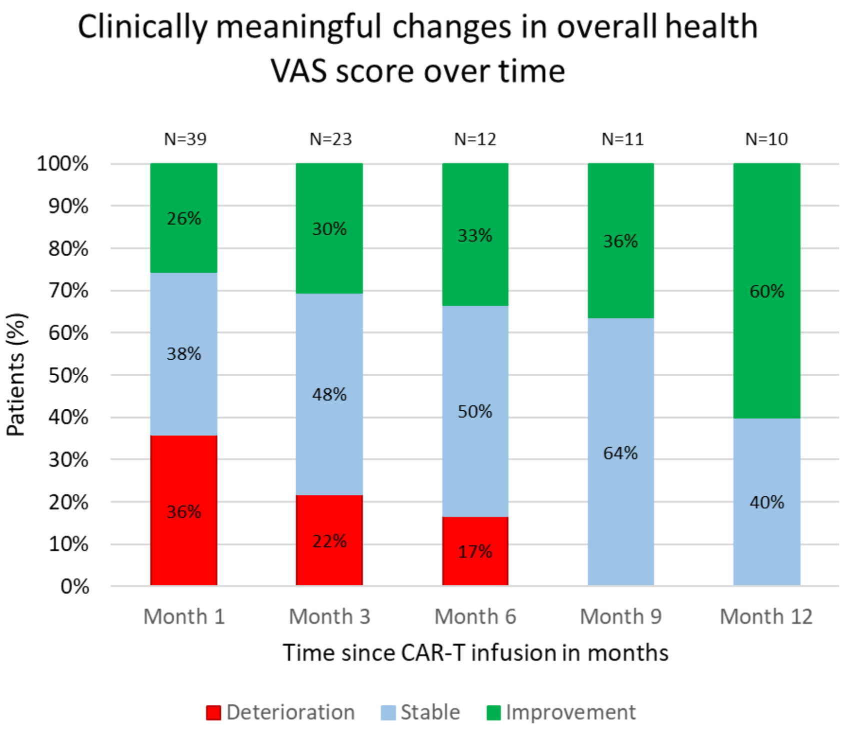

Supplementary S1

Dutch CAR-T Tumorboard eligibility criteria May 2020-May 2022

Histological diagnosis of large B-cell lymphoma (including diffuse large B-cell lymphoma, primary mediastinal B-cell lymphoma, high-grade B-cell lymphoma and transformed follicular lymphoma)

Relapsed/Refractory disease after at least 2 prior systemic therapy lines

No prior treatment with CD19-directed CAR-T therapy

No significant comorbidity, including poor performance status or organ dysfunction

Not disease that is too rapidly progressive

No central nervous system localization of lymphoma

Informed consent for CAR-T treatment

Supplementary S2

Efficacy outcomes of axi-cel in other real-world cohorts

	UK cohort[1]	Germany GLA/DRST cohort[2]	France DESCAR-T cohort[3]	Spain GETH/Geltamo cohort[4]	US-lymphoma cohort[5]	US CIBMTR cohort[6]
**N axi-cel infused**	224	173	209*	134	275	1297
**ORR (CR) axi-cel**	77% (52%)	74% (42%)	80% (60%)	60% (42%)	82% (64%)	73% (56%)
**OS median axi-cel**	15.6 months (95% CI: 11.1-NR)	NA	NR (14.7-NR)	13.9 months (95% CI: NA)	NR	21.8 (95% CI, 17.4 to 28.8)
**12-M OS rate axi-cel**	57.1% (95% CI: 49.8-63.8)	55% (95% CI: NA)	63.5% (95% CI: 55.0–70.8)	51% (95% CI: NA)	68% (95% CI: 63%-74%)	62% (95% CI, 60-65)
**PFS median axi-cel**	5.5 months (95% CI: 3.3-10.1)	NA	8.2 months (95% CI: 4.4-NA)	5.9 months (95% CI: NA)	8.3 months (95% CI: 6.0 - 15.1)	8.6 months (95% CI: 6.5-12.1)
**12-M PFS rate axi-cel**	41.8% (95% CI: 35.0-48.8)	35% (95% CI: NA)	46.6% (95% CI: 38.5–54.3)	41% (95% CI: NA)	47% (95% CI: 41-53)	47% (95% CI, 44% to 50%)

* Efficacy results available for axi-cel patients selected for matching according to propensity scored matching for tisa-cel versus axi-cel. Abbreviations: Axi-cel: axicabtagene ciloleucel, CI: confidence interval, CR: complete response, NA: data not available(i.e. not reported in publication), NR: not reached, ORR: overall response rate, OS: overall survival, PFS: progression-free survival

References

1. Kuhnl A, Roddie C, Kirkwood AA, Tholouli E, Menne T, Patel A, et al. A national service for delivering CD19 CAR-T in large B-cell lymphoma–The UK real-world experience. British Journal of Haematology. 2022;198(3):492-502.

1. Bethge WA, Martus P, Schmitt M, Holtick U, Subklewe M, von Tresckow B, et al. GLA/DRST real-world outcome analysis of CAR T-cell therapies for large B-cell lymphoma in Germany. Blood, The Journal of the American Society of Hematology. 2022;140(4):349-58.

3. Bachy E, Le Gouill S, Di Blasi R, Sesques P, Manson G, Cartron G, et al. A real-world comparison of tisagenlecleucel and axicabtagene ciloleucel CAR T cells in relapsed or refractory diffuse large B cell lymphoma. Nat Med. 2022;28(10):2145-54.

4. Kwon M, Iacoboni G, Reguera JL, Corral LL, Morales RH, Ortiz-Maldonado V, et al. Axicabtagene ciloleucel compared to tisagenlecleucel for the treatment of aggressive B-cell lymphoma. Haematologica. 2023.

5. Nastoupil LJ, Jain MD, Feng L, Spiegel JY, Ghobadi A, Lin Y, et al. Standard-of-Care Axicabtagene Ciloleucel for Relapsed or Refractory Large B-Cell Lymphoma: Results From the US Lymphoma CAR T Consortium. J Clin Oncol. 2020;38(27):3119-28.

6. Jacobson CA, Locke FL, Ma L, Asubonteng J, Hu ZH, Siddiqi T, et al. Real-World Evidence of Axicabtagene Ciloleucel for the Treatment of Large B Cell Lymphoma in the United States. Transplant Cell Ther. 2022;28(9):581.e1-
